# Hands-off: Feasibility and preliminary results of a two-armed randomized controlled trial of a web-based self-help tool to reduce problematic pornography use

**DOI:** 10.1556/2006.2021.00070

**Published:** 2021-10-29

**Authors:** Beáta Bőthe, Christian Baumgartner, Michael P. Schaub, Zsolt Demetrovics, Gábor Orosz

**Affiliations:** 1 Département de Psychologie, Université de Montréal, Montréal, Canada; 2 Institute of Psychology, ELTE Eötvös Loránd University, Budapest, Hungary; 3 Swiss Research Institute for Public Health and Addiction ISGF, Associated to the University of Zurich, Zurich, Switzerland; 4 Centre of Excellence in Responsible Gaming, University of Gibraltar, Gibraltar, Gibraltar; 5 Université d’Artois, Université de Lille, Université Littoral Côte d’Opale, ULR 7369 - URePSSS - Unité de Recherche Pluridisciplinaire Sport Santé Société, Atelier Sherpas, F-62800, Liévin, France

**Keywords:** cognitive-behavioral therapy, compulsive sexual behavior, feasibility, online intervention, problematic pornography use

## Abstract

**Background and Aims:**

Despite problematic pornography use (PPU) being prevalent, no previous study has examined the effectiveness of evidence-based interventions for PPU, using rigorous methods. Using a two-armed randomized controlled trial study design, we examined the feasibility and initial effectiveness of a six-week online PPU intervention.

**Methods:**

We recruited 264 participants (3.8% women, *M*_*age*_ = 33.2, *SD* = 10.6) who were randomized and assigned to either the self-help intervention (*n* = 123) or waitlist control condition (*n* = 141), and completed self-report questionnaires at baseline and after the end of the intervention (six-week follow-up). Multivariable linear regression models were generated and tested on a complete case basis to investigate possible treatment effects. Participants provided quantitative and qualitative feedback regarding the intervention’s content and appearance.

**Results:**

Participants evaluated all modules positively in the intervention in general. There were differential dropout rates (89.4% in intervention vs. 44.7% in control group) with an overall follow-up rate of 34.5%. The intervention group reported significantly lower levels of PPU (*P* < 0.001, *d* = 1.32) at the six-week follow-up. Moreover, they reported lower pornography use frequency (*P* < 0.001, *d* = 1.65), self-perceived pornography addiction (*P* = 0.01, *d* = 0.85), pornography craving (*P* = 0.02, *d* = 0.40), and higher pornography avoidance self-efficacy (*P* = 0.001, *d* = 0.87) at the six-week follow-up.

**Discussion and Conclusions:**

The present study was only a first step in rigorous treatment studies for PPU, but the findings are promising and suggest that online interventions for PPU might help reduce PPU in some cases, even without the guidance of therapists, by reducing treatment barriers.

## Introduction

Pornography use is prevalent among adolescents and adults (Bőthe, Vaillancourt-Morel, et al., 2020, 2021; Grubbs et al., 2020; Wright, Herbenick, & Paul, 2019). Findings of nationally representative studies from Australia (Rissel et al., 2017), Europe (Lewczuk, Glica, Nowakowska, Gola, & Grubbs, 2020), and the US (Grubbs, Kraus, & Perry, 2019; Herbenick et al., 2020) suggest that 84–94% of men and 54–87% of women report lifetime pornography use. Most individuals using pornography do not report distress or negative consequences deriving from their pornography use (Bőthe, Tóth-Király, Potenza, Orosz, & Demetrovics, 2020; Bőthe et al., 2021; Vaillancourt-Morel et al., 2017). Nevertheless, a small but significant ratio of people (1–3% of women and 4–11% of men) report problematic pornography use (PPU) (Grubbs et al., 2020; Grubbs, Kraus, & Perry, 2019; Rissel et al., 2017). PPU can be considered as a manifestation of Compulsive Sexual Behavior Disorder (CSBD) (Fernandez & Griffiths, 2019; Kafka, 2010), now included in the 11^th^ revision of the *International Statistical Classification of Diseases and Related Health Problems* (ICD-11, World Health Organization, 2019), and might be defined as uncontrollable, persistent patterns of pornography use despite personal distress and functional impairment in different areas of life (Bőthe, Tóth-Király, Demetrovics, & Orosz, 2021; Kraus et al., 2018).

Despite the prevalence of PPU and related treatment-seeking (Bőthe et al., 2021; Gola, Lewczuk, & Skorko, 2016; Kraus, Martino, & Potenza, 2016; Lewczuk, Szmyd, Skorko, & Gola, 2017), and proliferation of research in compulsive sexual behaviors and PPU in the past 25 years, there is still a virtual absence of rigorous, systematic, high-quality treatment-related research in PPU, resulting in the absence of effective treatment protocols for health care professionals and treatment-seeking individuals (Griffin, Way, & Kraus, 2021; Grubbs et al., 2020). Thus, the objective of the present study was to evaluate the feasibility and report the preliminary effectiveness of a new online intervention reducing PPU, using a two-armed randomized controlled trial (RCT) study design (Bőthe, Baumgartner, Schaub, Demetrovics, & Orosz, 2020; Rounsaville, Carroll, & Onken, 2001).

Despite the paucity of rigorous studies using gold-standard approaches (e.g., randomized controlled trials) to evaluate the effectiveness of interventions for CSBD and PPU (Efrati & Gola, 2018; Grubbs et al., 2020), some preliminary findings of previous treatment studies are available (e.g., Dhuffar & Griffiths, 2015; von Franqué, Klein, & Briken, 2015; Wéry & Billieux, 2017). However, it is important to note that most prior studies reported results of single case studies, used small, homogenous samples, and lacked proper assessment (i.e., validated measures) and control groups. The available scientific evidence regarding psychotherapeutic treatment options and their efficacy for PPU is largely limited.

Three studies reported the effectiveness of short interventions geared to reduce PPU (i.e., not compulsive sexual behaviors broadly) and included a control group (Crosby, 2011; Crosby & Twohig, 2016; Minarcik, 2016). These studies included elements of cognitive behavior therapy (CBT) and acceptance and commitment therapy (ACT), and each reported improvement in the participants’ pornography use-related symptoms. Although these studies demonstrated the potential efficacy and usefulness of CBT and ACT-based methods in reducing PPU, they were still limited by their small, homogenous samples (e.g., only men, only US samples), probably due to the interventions’ offline nature (i.e., individual sessions with a therapist). Thus, rigorous, inclusive investigations are necessary to move the field forward (Griffin et al., 2021; Grubbs et al., 2020; Klein, Savaş, & Conley, 2021).

Free, online interventions may overcome the aforementioned shortcomings, as they can reach larger and more diverse populations relatively easily, compared to traditional treatments, as was demonstrated in previous studies (Baumgartner et al., 2019; Haug, Castro, Wenger, & Schaub, 2018; Herrero et al., 2019; Weisel et al., 2018). Online interventions may also reduce other treatment barriers that can be present in offline interventions, such as unaffordability of traditional therapies, stigma, or feelings of shame for seeking help for PPU (Dhuffar & Griffiths, 2016). In addition, online interventions have already demonstrated their abilities to reduce other individual and social treatment barriers, given that they can be used any time privately the treatment-seeking individual feels the need for help, they are cost-effective or free, and easy-to-use (Baumgartner et al., 2019; Haug et al., 2018; Herrero et al., 2019; Weisel et al., 2018). Despite recent calls for randomized controlled trials to examine the effectiveness of interventions for PPU (Griffin et al., 2021; Grubbs et al., 2020) and the advantages of online interventions, compared to traditional interventions, no prior study examined the efficacy of online interventions for PPU.

As a first step in the field, the present study aimed to document the feasibility and initial effectiveness of an online self-help program to reduce PPU, considering previous recommendations (Arain, Campbell, Cooper, & Lancaster, 2010; Bowen et al., 2009; Orsmond & Cohn, 2015; Rounsaville et al., 2001). To provide preliminary findings of the effectiveness of the intervention, as the primary outcome, we examined the change in participants’ PPU between baseline and six-week follow-up assessments, compared to a waitlist control condition. We hypothesized that participants in the intervention condition would report a decrease, while participants in the control condition would not report significant changes in their PPU over time. The secondary outcome measures included positive changes in participants’ pornography use frequency, self-reported addiction, cravings, moral incongruence, time spent with pornography per session, and self-efficacy to avoid pornography use. We hypothesized that participants in the intervention condition would report beneficial changes in the secondary outcomes over time, while participants in the control condition would not report significant changes over time.^1^

## Methods

### Study design

This study used a two-armed randomized controlled trial study design, examining the feasibility and potential efficacy of a PPU online intervention, with a waitlist control condition and follow-up assessments right after the end of the six-week-long intervention. Participants were randomized after completing the baseline measures at an individual level by an automated, computer-based algorithm on the intervention website with a randomization list (1:1 ratio). Participants were informed about their condition assignment, but they were not aware of the study’s hypotheses. Any blinding of study personnel was unwarranted, as they were not directly involved in the intervention. Participants in the intervention condition were granted immediate access to the intervention. Participants in the control condition received access to the intervention three months after completing the baseline measures. CONSORT guidelines were followed (see Appendix 1), the study was preregistered before starting the recruitment (https://osf.io/5tqkb), and a detailed study protocol was already published (Bőthe, Baumgartner, et al., 2020).

### Procedure and participants

At the beginning of the study, we aimed to recruit people from Switzerland and Hungary mainly. However, participants from other countries were also invited and included in the study. Based on a priori sample size calculation with 80% power to detect small differences with an alpha error of 5% and two-tailed testing (Bőthe, Baumgartner, et al., 2020), a minimum sample size of 242 participants (121 participants in each group) was deemed sufficient to detect differences between the intervention and control groups over time. We recruited a total of 361 participants on psychology news websites (e.g., PsyPost), social media sites (e.g., Reddit), and Google advertisements between February 2019 and December 2020, as the target sample size was reached.

**Fig. 1.**
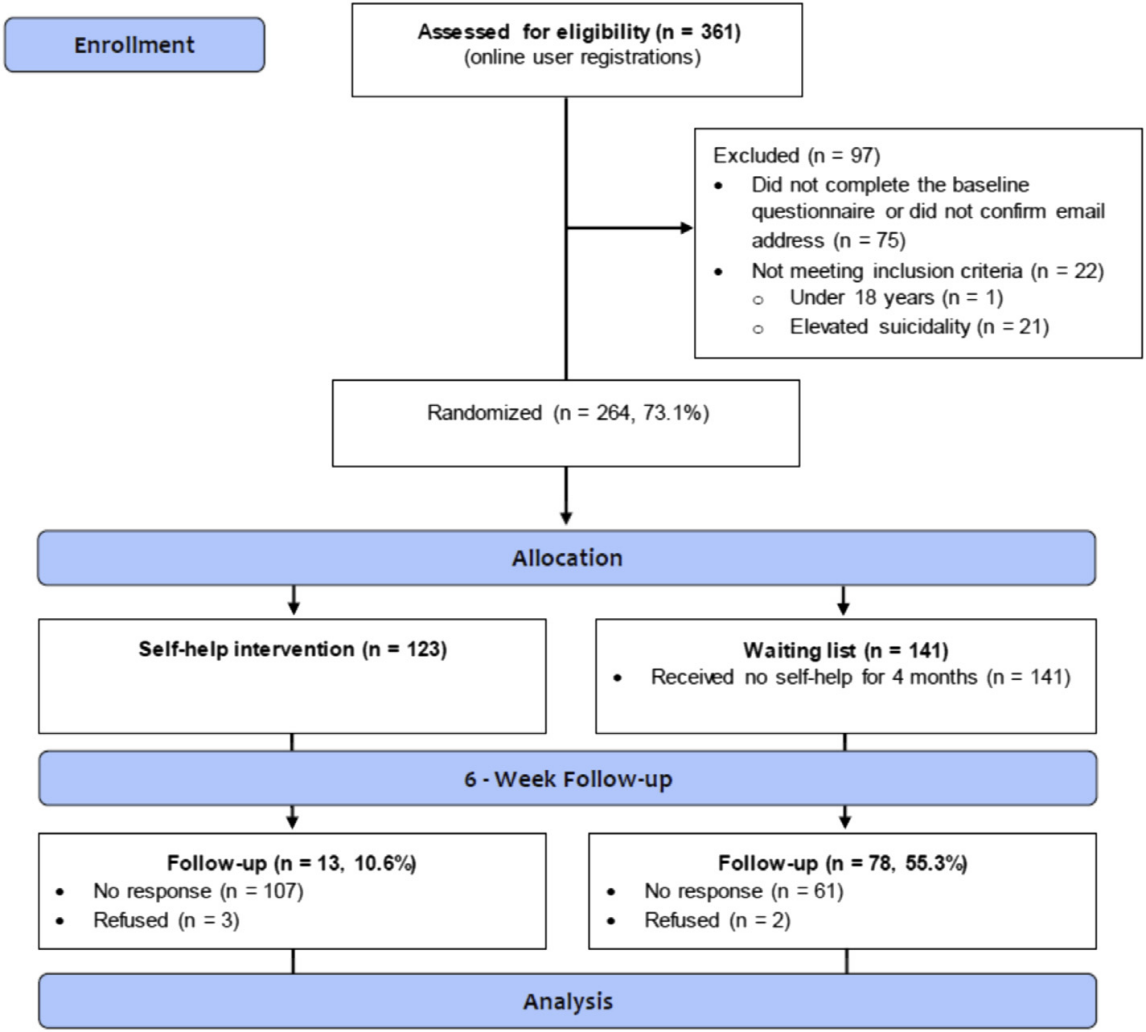
Flowchart of Participants Based on the CONSORT Criteria *Note.* CONSORT=Consolidated Standards of Reporting Trials.

Individuals who were 18 years old or older; had sufficient skills in the English language in reading and writing; had a valid email address and internet access at least for one hour each week; read the informed consent; and agreed to participate were included in the study. Participants were excluded from the study if they did not have a valid email address or did not complete the baseline questionnaire (22 participants). The final sample consisted of 264 participants (3.8% women, *M*_*age*_ = 33.2, *SD* = 10.6). Most participants had a college or university degree (77.3%), and the majority of them resided in the United States (37.9%), England (15.5%), or Canada (7.6%). A total of 73.5% of the participants were heterosexual, and approximately half of them were in a romantic relationship. The detailed participant flow is shown in Fig. 1, and participants’ sociodemographic characteristics are shown in Table 1.

**Table 1.** Baseline sociodemographic, pornography use-related, and psychological characteristics of participants in the intervention and control groups

	Intervention group (*n =* 123)	Waitlist control group (*n =* 141)	Total (*N =* 264)	Statistical analysis *(Chi-Square Test, ANOVA)*
*Gender*, *n* (%)				*χ* ^2^ (2, *N* = 264) = 0.00, *P* = 1.000
Woman	5 (4.1)	5 (3.5)	10 (3.8)	
Man	118 (95.9)	136 (96.5)	254 (96.2)	
*Age*, *M (SD)*	33.3 (11.5)	33.1 (9.9)	33.2 (10.6)	*F* (2,262) = 0.02, *P* = 0.876
*Highest education*, *n* (%)				*χ* ^2^ (3, *N* = 264) = 1.82, *P* = 0.612
Primary school	0 (0.0)	2 (1.4)	2 (0.8)	
Vocational school	2 (1.6)	2 (1.4)	4 (1.5)	
High school	26 (21.1)	28 (19.9)	54 (20.5)	
College or university	95 (77.2)	109 (77.3)	127 (77.3)	
*Country of origin*, *n* (%)				*χ* ^2^ (5, *N* = 264) = 3.71, *P* = 0.592
United States	43 (35.0)	57 (40.4)	100 (37.9)	
England	19 (15.4)	22 (15.6)	41 (15.5)	
Canada	12 (9.8)	8 (5.7)	20 (7.6)	
Hungary	10 (8.1)	8 (5.7)	18 (6.8)	
India	3 (2.4)	7 (5.0)	10 (3.8)	
Other (combined^a^)	36 (29.2)	39 (27.7)	75 (28.4)	
*Relationship status*, *n* (%)				*χ* ^2^ (5, *N* = 264) = 10.01, *P* = 0.075
Single	43 (35.0)	68 (48.2)	111 (42.0)	
In a relationship	40 (32.5)	30 (21.3)	70 (26.5)	
Married	37 (30.1)	34 (24.1)	71 (26.9)	
Engaged	2 (1.6)	6 (4.3)	8 (3.0)	
Divorced	0 (0.0)	2 (1.4)	2 (0.8)	
Other	1 (0.8)	1 (0.7)	2 (0.8)	
*Sexual orientation, n* (%)				*χ* ^2^ (3, *N* = 264) = 4.58, *P* = 0.205
Heterosexual	92 (74.8)	102 (72.3)	194 (73.5)	
Homosexual	7 (5.7)	6 (4.3)	13 (4.9)	
Bisexual	17 (13.8)	30 (21.3)	47 (17.8)	
Unsure	7 (5.7)	3 (2.1)	10 (3.8)	
*Sought treatment for pornography use previously, n* (%)	40 (32.5)	48 (34.0)	88 (33.3)	*χ* ^2^ (1, *N* = 264) = 0.02, *P* = 0.896
*Problematic pornography use* (Range 0–126), *M (SD)*	80.5 (16.6)	81.1 (20.4)	80.8 (18.7)	*F* (1,262) = 0.06, *P* = 0.812
*Pornography use frequency*, *n* (%)				*χ* ^2^ (5, *N* = 264) = 0.09, *P* = 0.753
>7 a week	35 (28.5)	44 (31.3)	79 (29.9)	
6–7 a week	21 (17.1)	20 (14.2)	41 (15.5)	
4–5 a week	22 (17.9)	25 (17.7)	47 (17.8)	
2–3 a week	19 (15.4)	24 (17.0)	43 (16.3)	
weekly	11 (8.9)	16 (11.3)	27 (10.2)	
Less frequently	15 (12.4)	12 (8.5)	27 (10.2)	
*Time spent with pornography use per session in minutes*, *M (SD)*	50.9 (47.3)	53.4 (52.2)	52.2 (50.47)	*F* (1,261) = 0.15, *P* = 0.696
*Moral incongruence concerning pornography use* (Range 0–6), *M (SD)*	3.0 (2.1)	3.3 (2.2)	3.2 (2.2)	*F* (1,262) = 0.87, *P* = 0.351
*Self-perceived pornography addiction* (Range 0–6), *M (SD)*	4.6 (1.5)	4.7 (1.4)	4.7 (1.4)	*F* (1,262) = 0.12, *P* = 0.729
*Pornography craving* (Range 0–60), *M (SD)*	46.7 (15.9)	47.0 (15.6)	46.9 (15.7)	*F* (1,262) = 0.04, *P* = 0.840
*Pornography avoidance self-efficacy* (Range 0–100), *M (SD)*	52.2 (18.0)	51.8 (18.9)	52.0 (18.5)	*F* (1,262) = 0.04, *P* = 0.834
*Sex mindset*, (Range 5–30), *M (SD)*	13.0 (5.2)	13.7 (5.1)	13.4 (5.2)	*F* (1,262) = 1.12, *P* = 0.292
*Sexual satisfaction,* (Range 0–4), *M (SD)*	2.1 (1.3)	2.0 (1.2)	2.0 (1.3)	*F* (1,149) = 0.25, *P* = 0.617
*Satisfaction with life,* (Range 5–35), *M (SD)*	18.9 (7.5)	17.2 (7.6)	18.0 (7.6)	*F* (1,262) = 3.31, *P* = 0.070
*Self-report adult ADHD* ^b^ (Range 0–24), *M (SD)*	12.0 (4.2)	12.1 (4.5)	12.0 (4.4)	*F* (1,262) = 0.05, *P* = 0.827
*Alcohol-related problems*, (Range 0–45), *M (SD)*	2.6 (5.9)	2.6 (4.4)	2.6 (5.1)	*F* (1,262) = 0.00, *P* = 0.973
*Psychiatric symptoms (depressive, anxiety, and somatization symptoms)* (Range 0–72), *M (SD)*	18.3 (12.5)	20.0 (11.5)	19.2 (12.0)	*F* (1,262) = 1.23, *P* = 0.268
*Positive emotions,* (Range 0–20), *M (SD)*	15.0 (3.5)	14.9 (4.3)	14.9 (4.0)	*F* (1,262) = 0.08, *P* = 0.780
*Negative emotions,* (Range 0–20), *M (SD)*	17.3 (4.0)	17.2 (4.6)	17.2 (4.3)	*F* (1,262) = 0.04, *P* = 0.845
*Cannabis use* ^*c*^, *n* (%)				*χ* ^2^ (4, *N* = 264) = 8.01, *P* = 0.091
Never	84 (68.3)	94 (66.7)	178 (67.4)	
Once or twice	20 (16.3)	14 (9.9)	34 (12.9)	
Monthly	4 (3.3)	8 (5.7)	12 (4.5)	
Weekly	9 (7.3)	7 (5.0)	16 (6.1)	
Daily or almost daily	6 (4.9)	16 (12.8)	24 (9.1)	
*Prescription stimulants use* ^*c*^, *n* (%)				*χ* ^2^ (4, *N* = 264) = 0.54, *P* = 0.464
Never	104 (84.6)	114 (80.9)	218 (82.6)	
Once or twice	2 (1.6)	2 (1.4)	4 (1.5)	
Monthly	0 (0.0)	0 (0.0)	0 (0.0)	
Weekly	1 (0.8)	5 (3.5)	6 (2.3)	
Daily or almost daily	16 (13.0)	20 (14.2)	36 (13.6)	

*Note*. ^a^Less than 5 participants in each unlisted country, ^b^ADHD: attention deficit hyperactivity disorder, ^c^Previous three months; the two most commonly used substances (i.e., cannabis and prescription stimulants) are included in table. *M* = mean, *SD* = standard deviation.

### Intervention

An automated, web-based intervention (Hands-off; www.hands-off.net) was developed by the authors based on the principles of motivational interviewing (Rollnick & Miller, 1995), cognitive-behavioral therapy (Meichenbaum, 1977), mindfulness techniques (Altman, 2014), “wise” social-psychological interventions (Yeager & Walton, 2011; Walton, 2014; Walton & Wilson, 2018), and previous online interventions developed by the Swiss Research Institute for Public Health and Addiction that effectively reduced substance use, alcohol use, and problematic gambling (Baumgartner et al., 2019; Schaub et al., 2013, 2016). The intervention included six modules and one booster module. The six core modules were required to be completed in their intended order, and the booster module was available for participants to be completed one month after finishing the sixth module. Participants were instructed to complete one module each week and were encouraged to repeat any modules they found helpful. Modules’ completion took between 45 and 60 min on average.

The first module gave a general overview of the intervention and motivated participants to reflect on their pornography use. The second module helped participants identify risk situations that might result in pornography use and taught strategies for dealing with them. The third module reflected on how to change pornography use habits and integrate pleasurable activities into participants’ everyday lives. The fourth module concentrated on identifying triggers for cravings and taught strategies to reduce cravings. The fifth module introduced automatic negative thoughts and frequent common thinking errors, and strategies to challenge automatic negative and develop balanced thoughts. The sixth module reviewed the previous modules’ content and participants’ achievements and helped them plan strategies to prevent relapses. The booster module reviewed participants’ past four weeks and provided participants the opportunity to review their past months and plan to preserve success in the long run. In addition, four fictional companions representing typical problematic pornography users were included in the modules to encourage reflection on specific questions.

Besides the intervention modules, the program included a dashboard which was the main page of the intervention. It provided helpful information for participants (e.g., dates of the follow-up assessments, activity planner, access to pornography use diary). A daily diary was also embedded on the website, assessing participants’ targeted and actual pornography use frequency per day and mood. A personal graph was presented for participants for visual feedback. Safety measures (e.g., emergency contacts) and other elements (e.g., participants could revisit some of their inputs to specific questions in the intervention modules) were included in the intervention.

Participants in the waitlist control condition were provided the opportunity to participate in the intervention three months after completing the baseline survey. Similarly to participants in the intervention condition, participants in the control condition also completed baseline and follow-up measures.

### Measures

#### Primary outcome

The primary outcome was self-reported scores on the Problematic Pornography Consumption Scale (PPCS) (Bőthe, Tóth-Király, et al., 2018). The PPCS assesses PPU with 18 items; participants indicated their answers on seven-point scales (1 = “never”; 7 = “all the time”; *α* = 0.90). A score of 76 (out of 126) or higher indicates a high risk of PPU.

#### Secondary outcomes

Pornography use frequency^2^, duration of pornography use per each session (Bőthe, Bartók, et al., 2018; Bőthe et al., 2021), perceived addiction and moral incongruence regarding pornography use (Grubbs, Kraus, & Perry, 2019). Moreover, the 12-item Pornography Craving Questionnaire (PCQ; *α* = 0.88) (Kraus & Rosenberg, 2014), and the 18-item Pornography-Use Avoidance Self-Efficacy Scale (PASS; *α* = 0.90) (Kraus, Rosenberg, Martino, Nich, & Potenza, 2017) were assessed as secondary outcomes in this study.

#### Other variables

Participants’ sociodemographic information (e.g., gender, age, relationship status, and sexual orientation), sexuality-related questions (e.g., number of lifetime sexual partners), and previous treatment-seeking for PPU were assessed at baseline (Bőthe, Bartók, et al., 2018; Bőthe et al., 2021).

The one-item sexual satisfaction measure (Mark, Herbenick, Fortenberry, Sanders, & Reece, 2014), the five-item Sex Mindset Scale (SMS; *α* = 0.81) (Bőthe, Tóth-Király, Demetrovics, & Orosz, 2017), the five-item Satisfaction with Life Scale (SWLS; *α* = 0.89) (Diener, Emmons, Larsen, & Griffin, 1985), and the ten-item Positive and Negative Affect Scale (PANAS; *α*_positive_ = 0.43, *α*_negative_ = 0.44) (Gyollai, Simor, Köteles, & Demetrovics, 2011) were measured. The 18-item Brief Symptom Inventory (BSI-18; *α* = 0.89) (Asner-Self, Schreiber, & Marotta, 2006), the six-item Adult ADHD Self-report Screening Scale for DSM-5 (ASRS-5; *α* = 0.72) (Ustun et al., 2017), the ten-item NIDA Assist (NIDA) (Group, 2002), the 15-item Alcohol-Related Problems: Short Inventory of Problems (SIP; *α* = 0.94) (Miller, Tonigan, & Longabaugh, 1995), the five-item P4 Suicidality Screener (P4) (Dube, Kroenke, Bair, Theobald, & Williams, 2010) were included in the study.

Moreover, after completing each module, participants were asked to rate the usefulness, understandability, length, appearance, likelihood of quitting the module before finishing the module, likelihood of quitting the program after finishing the module, and provide an overall rating. Participants rated each statement on a 0–100% scale. Higher scores indicate more positive attitudes in the case of usefulness, understandability, appearance, and overall rating. In the case of length and quitting questions, higher scores indicate more negative attitudes. Participants could also add qualitative feedback after finishing each module concerning the most and least useful parts and exercises, what they would change in the module, and write any other comments they had. For measurement details, see the study protocol (Bőthe, Baumgartner, et al., 2020).

### Statistical analyses

Although we originally planned to analyze the data on an intention-to-treat basis (Bőthe, Baumgartner, et al., 2020), we could not conduct the analysis due to high attrition (see Fig. 1). Therefore, we used a treatment-of-the-treated, complete case analysis focusing on the pre-intervention and post-intervention follow-up data six weeks after baseline (i.e., six-week follow-up). Descriptive statistics were computed in R version 3.6.1 (R Core Team, 2019). To compare the baseline characteristics of participants in the control and intervention groups, we conducted chi-square tests and ANOVAs, depending on the outcome variable (i.e., categorical, ordinal, or continuous). For investigating possible treatment effects, multivariable linear regression models were generated and tested on a complete case basis, using the package “stats” in R. Change scores between baseline and 6-week follow-up served as dependent variables for the outcomes, with study condition set as the independent variable. Each outcome was adjusted for its baseline value.

### Ethics

The authors assert that all procedures contributing to this work comply with the ethical standards of the relevant national and institutional committees on human experimentation and with the Helsinki Declaration. The present research was approved by the Institutional Ethical Review Board of the Eötvös Loránd University (2018/249-2). All participants were informed about the study, and all provided informed consent.

## Results

### Participation flow, and overall and differential attrition

Figure 1 overviews the trial flow. Between February 2019 and December 2020, 361 potential participants were recruited, of which 26.9% were ineligible to participate in the study or did not complete the baseline questionnaire. Therefore, 264 participants were randomized and assigned to either the self-help intervention (*n* = 123) or the waitlist control condition (*n* = 141). The six-week follow-up was completed by 91 participants (34.5% of the initial sample). There were significant differences in the dropout rates between the intervention and control groups, with only 11% of the intervention group completing the six-week follow-up compared to 55% in the control group (χ^2^(1, *N* = 264) = 56.27, *P* < 0.001). We further examined overall attrition (independent of condition) and differential attrition (dependent on condition). Concerning overall attrition, participants retained in both conditions, as compared to those not retained, did not have significantly different demographic and psychological characteristics, except for satisfaction with life (*t* (197.4) = -2.07, *P* = 0.039). Participants dropping out from the study reported significantly higher satisfaction with their life at baseline (*M* = 18.68, *SD* = 7.76) than those retained (*M* = 16.70, *SD* = 7.12). Concerning differential attrition, no significant differences were observed between those who dropped out and completed the follow-up measurement in the control group (all *ps* > 0.180). Moreover, no significant differences were revealed between those who completed vs. did not complete the follow-up assessment in the intervention group, except for pornography craving (*t* (17.7) = -2.45, *P* = 0.025). Participants dropping out from the intervention reported a significantly higher pornography craving score at baseline (*M* = 47.59, *SD* = 16.07) than those retained (*M* = 38.69, *SD* = 11.86).

### Participants’ characteristics

Concerning the primary outcome, participants reported high levels of PPU (*M* = 80.8, *SD* = 33.3), which is above the suggested cut-off score (i.e., 76), and one-third of the participants had sought treatment for their pornography use before. Most participants used pornography regularly, with 30% of them using it more than seven times a week, and on average, they spent almost an hour (*M* = 52 min, *SD* = 50) with pornography use per session. Participants reported high levels of self-perceived pornography addiction (*M* = 4.7, *SD* = 1.4), pornography craving (*M* = 46.9, *SD* = 15.7), and low levels of pornography avoidance self-efficacy (*M* = 52.0, *SD* = 18.5). No significant differences were observed between the intervention and control group concerning their baseline sociodemographic, pornography use-related, and psychological characteristics. Participants’ detailed characteristics can be seen in Table 1.

Given the high attrition rate in the intervention group, we pushed forward the comparison of participants’ characteristics and examined potential differences between participants in the intervention group who did not complete any modules (*n* = 41), completed only one module (*n* = 37), and completed more than one module (*n* = 45) (Appendix 2, Table A1.) No significant differences were observed between these groups, except for age. Participants were significantly younger in the group of those who only completed one module (*M* = 28.4, *SD* = 8.8), compared to those who did not complete any modules (*M* = 35.4, *SD* = 11.7) and completed more than one module (*M* = 35.4, *SD* = 12.2).

### Results of preliminary analysis and efficacy of the intervention

Based on the complete case analysis (Tables 2 and 3), participants in the intervention group, compared to the control group, reported significantly lower levels of PPU (*B* = –19.33; CI = –28.75, –9.91; *P* < 0.001, *d* = 1.32). In the intervention group, participants’ PPU scores were under the suggested cut-off score (76 points) at the six-week follow-up (*M* = 64.00, *SD* = 14.81), while the control group participants’ PPU scores were still above the cut-off (*M* = 80.06, *SD* = 20.77). Similarly, pornography use frequency was significantly lower in the intervention group (*M* = 2.44, *SD* = 1.69) at the six-week follow-up (*B* = –2.88; CI = –4.11, –1.65; *P* < 0.001, *d* = 1.65), while participants’ pornography use frequency remained unchanged in the control group (*M* = 5.12, *SD* = 2.35). Compared to the control group, participants in the intervention group reported lower self-perceived pornography addiction (*B* = –1.04; CI = –1.83, –0.24, *P* = 0.010, *d* = 0.85) and higher pornography avoidance self-efficacy at the six-week follow-up (*B* = 17.89; CI = 7.22, 28.56, *P* = 0.001, *d* = 0.87), and significantly lower levels of pornography craving (*B* = –10.45; CI = –18.91, –1.99, *P* = 0.020, *d* = 0.40). However, participants’ moral incongruence toward pornography use and time spent with pornography use on each session did not change significantly (*B* = 0.12; *P* = 0.530).

**Table 2.** Results of the complete case analysis concerning pornography use-related variables

Variables	6-week follow-up		
	B (95% CI)	*β* (95% CI)	*P*
Problematic pornography use	−19.33 (−28.75, −9.91)	−1.12 (−1.67, −0.58)	<0.001
Pornography craving	−10.45 (−18.91, −1.99)	−0.71 (−1.28, −0.13)	0.02
Moral incongruence concerning pornography use	0.61 (−0.17, 1.40)	0.47 (−0.14, 1.09)	0.12
Self-perceived pornography addiction	−1.04 (−1.83, −0.24)	−0.66 (−1.16, −0.15)	0.01
Pornography avoidance self-efficacy	17.89 (7.22, 28.56)	0.95 (0.39, 1.53)	0.001
Pornography use frequency last 7 days	−2.88 (−4.11, −1.65)	−1.44 (−2.06, −0.83)	<0.001
Time spent with pornography use per session in minutes	−7.75 (−32.16, 16.66)	−0.16 (−0.66, 0.34)	0.53

*Note*. 95% CI = 95% confidence interval.

**Table 3.** Comparison of the control and intervention groups concerning pornography use-related variables based on the complete case analysis

	Waitlist control group (*n* = 73)		Intervention group (*n* = 13)		
Variables	Baseline *M (SD)*	6-week follow-up *M (SD)*	Baseline *M (SD)*	6-week follow-up *M (SD)*	Cohen's *d* ^a^ (95% CI)
Problematic pornography use	79.63 (20.30)	80.06 (20.77)	85.31 (19.43)	64.00 (14.81)	1.32 (0.68, 1.92)
Pornography craving	46.58 (16.64)	47.40 (15.80)	38.69 (11.86)	31.64 (12.19)	0.40 (−0.20, 0.98)
Moral incongruence concerning pornography use	3.29 (2.20)	3.18 (2.18)	2.85 (2.03)	3.63 (1.96)	−0.51 (−1.09, 0.10)
Self-perceived pornography addiction	4.67 (1.47)	4.90 (1.31)	4.92 (0.86)	4.00 (1.34)	0.85 (0.24, 1.44)
Pornography avoidance self-efficacy	52.54 (19.77)	51.63 (20.75)	56.32 (17.90)	73.08 (17.55)	−0.87 (−1.46, −0.25)
Pornography use frequency in the last 7 days	4.94 (2.44)	5.12 (2.35)	5.36 (2.17)	2.44 (1.69)	1.65 (0.99, 2.26)
Time spent with pornography use per session in minutes	56.81 (62.21)	54.16 (53.48)	37.85 (40.49)	36.54 (34.00)	−0.02 (−0.61, 0.56)

*Note*. ^a^Cohen's *d* reflects the difference between the intervention and control groups based on the change scores (i.e., from baseline to 6-week follow-up). 95% CI = 95% confidence interval. *M* = mean, *SD* = standard deviation.

**Table 4.** Participants feedback on the usefulness, length, overall rating of the modules

Modules^a,b^	Usefulness *M (SD)* ^*c*^	Understandability *M (SD)* ^*c*^	Length *M (SD)* ^*c*^	Appearance *M (SD)* ^*c*^	Likelihood of quitting the module before finishing the module *M (SD)* ^*c*^	Likelihood of quitting the program after finishing the module *M (SD)* ^*c*^	Overall rating *M (SD)* ^*c*^
*Module 1:* Introduction and the possibility of change (*n* = 120)	74.58 (22.82)	86.92 (17.72)	28.75 (30.61)	81.33 (22.52)	25.50 (26.05)	16.25 (22.61)	78.17 (20.94)
*Module 2:* Why do I watch porn, and how can I change it? Identifying internal and external risk situations (*n* = 54)	78.15 (24.58)	84.07 (19.48)	22.78 (26.16)	78.89 (22.88)	23.89 (28.38)	11.48 (17.20)	77.59 (27.40)
*Module 3:* How to feel better without porn? (*n* = 34)	78.82 (18.38)	87.94 (14.73)	33.82 (29.13)	85.59 (14.81)	26.76 (27.93)	16.47 (24.73)	73.82 (30.55)
*Module 4:* What can I do about my cravings? (*n* = 22)	82.27 (20.45)	82.73 (20.04)	15.00 (24.83)	85.00 (18.20)	13.18 (19.85)	9.09 (16.01)	78.18 (28.89)
*Module 5:* Behaviors, Emotions, and Thoughts (BET): I BET you can do it (*n* = 18)	77.22 (31.59)	81.11 (22.72)	15.56 (22.29)	81.11 (22.72)	11.67 (24.55)	3.33 (5.90)	79.44 (26.89)
*Module 6:* How to preserve your success? (*n* = 15)	82.67 (18.70)	94.00 (9.86)	8.00 (17.40)	88.67 (14.57)	6.00 (18.05)	6.67 (20.59)	82.00 (27.83)

*Note.* ^a^The Modules column of the table has been published in the study protocol paper (Bőthe, Baumgartner, et al., 2020). ^b^As only 5 participants completed the Booster module (module 7), and it took place four weeks after finishing the intervention, we did not include it in the present analysis. The number of participants who provided feedback for each module is presented in parentheses after each module's title. As participants in the waitlist control condition were provided the opportunity to participate in the intervention three months after completing the baseline survey, their responses, and active participants' responses are also included in this table for comprehensiveness, resulting in larger subsample sizes than number of participants completing each module in the intervention group in Fig. 2. ^c^The range of response options for all questions was between 0% and 100%. Higher scores indicate more positive attitudes in the case of usefulness, understandability, appearance, and overall rating. In the case of length and quitting questions, higher scores indicate more negative attitudes. As 0 was the default value for all questions, we removed those participants' data from this analysis who had a 0 answer for all questions. *M* = mean, *SD* = standard deviation.

### Adherence and participants’ feedback on the intervention

**Fig. 2.**
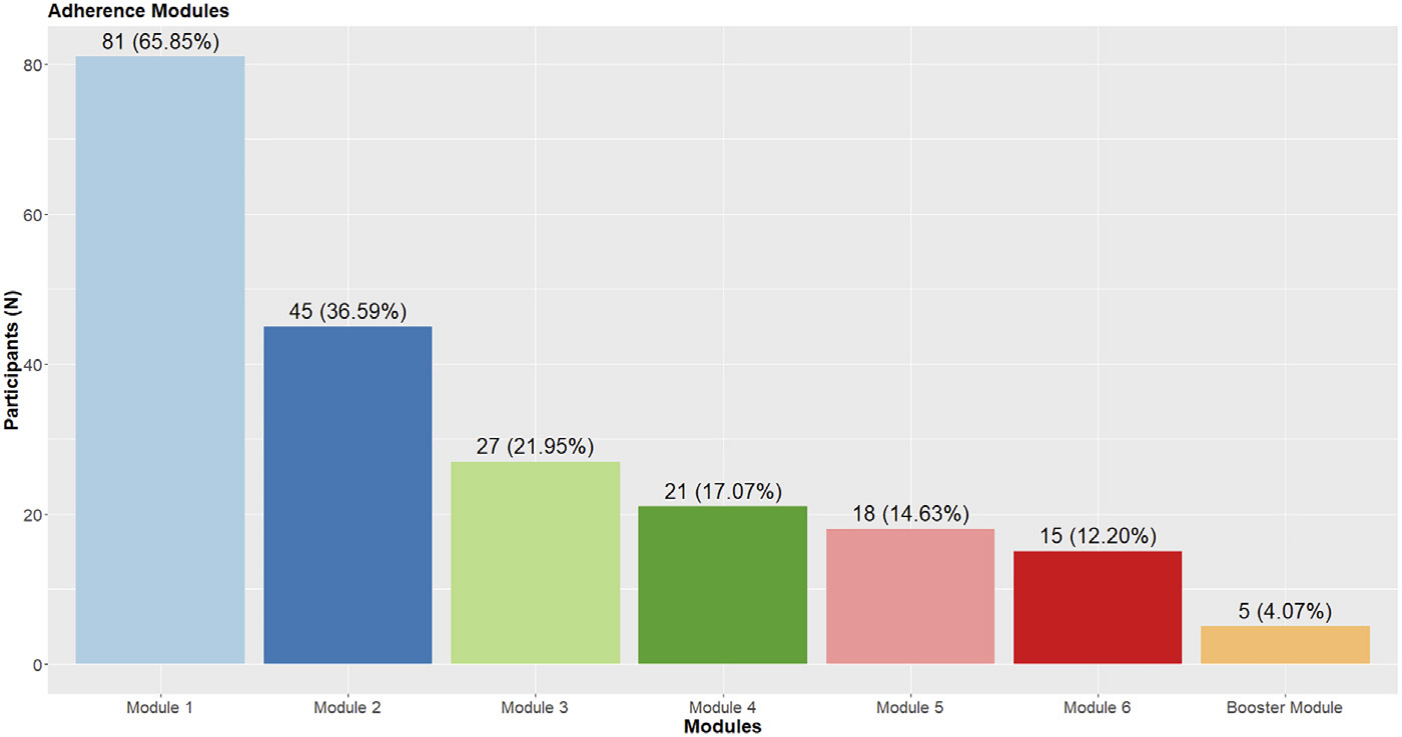
Completion of modules in the intervention group

Two-thirds of the participants in the intervention group completed the first module of the intervention, while only 12% of them completed the sixth module, with a gradual decrease in completion (see Fig. 2). On average, participants completed 1.73 modules (*SD* = 2.07). Participants evaluated all modules positively in the intervention in general (*M* = 73.82–82.00). Similarly high scores were reported concerning the modules’ usefulness (*M* = 74.58–82.67), understandability (*M* = 81.11–94.00), and appearance (*M* = 78.89–88.67). Participants reported high satisfaction with the modules’ length (*M* = 8.00–33.82) and a low likelihood of quitting before finishing the given module (*M* = 6.00–26.76) or quitting the program after finishing the module (*M* = 3.33–16.47) (Table 4). Qualitative feedback corroborated participants’ quantitative answers (Table 5). Participants mentioned finding useful those components of given modules that were aimed to be addressed (e.g., identification of advantages and disadvantages of pornography use in module 1). Nevertheless, participants also mentioned some components and characteristics of the intervention (e.g., not having relatively old companions) that should be changed. Moreover, some tasks were considered useful by some participants, while others considered them less useful (e.g., advising a companion in module 2).

**Table 5.** Modules in the intervention and summary of participants' qualitative feedback

Modules^a,b^	Content^a^	Components mentioned as most useful^c^	Components mentioned as least useful^c^
*Module 1:* Introduction and the possibility of change (*n* fffff 120)	- General overview - Introduction to fictional companions - Reflections on personal pornography use (e.g., advantages and disadvantages, reasons for change, reviewing useful resources for a change)	- Reflecting on past success - Identification of pros and cons of pornography use - Identification of reasons to quit pornography use - Looking at the antecedents and consequences of pornography use all at one time on the page - Companions and reading about their experiences - Interactive parts - Diary, identifying resources that already have	- Identification of pros and cons of pornography use - Identification of resources - Age (i.e., only relatively young companions) and credibility of companions - High number of questions and typing tasks - Too much reading and text - Too generic examples - Some components seem redundant - More explanation is needed for some tasks - No human interaction - Listing habits the participant has already changed
*Module 2:* Why do I watch porn, and how can I change it? Identifying internal and external risk situations (*n* = 54)	- Identification of the internal and external risk situations that can lead to pornography use - Learning how to deal with these risk situations	- Mindfulness - Listing the feelings followed by porn use - Examples of companions - Identification and reflection on triggers - The break-down of how to tackle risk situations - Giving advice to a companion	- Giving advice to a companion - Giving advice to future self - Explaining mindfulness in own words, “too academical” examples (e.g., linked blogs), and already knowing about mindfulness - Age (i.e., only relatively young companions) of companions - Not mentioning "masturbation addiction" - Some figures were too small on phones
*Module 3:* How to feel better without porn? (*n* = 34)	- Learning how to change personal pornography using habits - Learning how to integrate joyful activities into everyday life	- Identifying things to do instead of pornography use (fun activities) - Bringing up earlier responses from the participant about the reasons for quitting pornography use - The realization that good habits can become lasting habits given time - Knowledge that willpower can be changed	- List of sports and free time activities
*Module 4:* What can I do about my cravings? (*n* = 22)	- Identification of personal triggers for cravings - Learning strategies to reduce craving	- Learning about how cravings work (e.g., visualizations) - Learning about how to deal with cravings	- Bringing up earlier responses from the participant - Giving advice for future participants
*Module 5:* Behaviors, Emotions, and Thoughts (BET): I BET you can do it (*n* = 18)	- Getting to know automatic negative thoughts and the most frequent common thinking errors - Learning about the relations between one's thoughts, emotions, and pornography use - Learning strategies to challenge automatic negative thoughts and develop balanced thoughts	- Learning about automatic negative thoughts - Explanations and examples - Learning about how to deal with automatic negative thoughts	- Keeping a thought diary (pdf) on a shared computer might be problematic
*Module 6:* How to preserve your success? (*n* = 15)	- Reviewing the main contents of the previous modules - Identification of one's toughest moments in the program and how he/she overcame them - Planning strategies to prevent relapses to previous pornography use habits	- Reviewing previous content and opportunity to go back to earlier modules - Best strategies part - Prevention plan	- NA

*Note.* ^a^The Modules and Content columns of the table have been published in the study protocol paper (Bőthe, Baumgartner, et al., 2020). ^b^As only 5 participants completed the Booster module (module 7), and it took place four weeks after finishing the intervention, we did not include it in the present analysis. ^c^As the number of participants gradually decreased, the number of and variety of mentioned topics in the feedback also decreased. The number of participants who provided qualitative feedback for each module is presented in parentheses after each module's title. As participants in the waitlist control condition were provided the opportunity to participate in the intervention three months after completing the baseline survey, their responses, and active participants' responses are also included in this table for comprehensiveness, resulting in larger subsample sizes than number of participants completing each module in the intervention group in Fig. 2.

## Discussion

Despite PPU being prevalent, no previous study has examined the effectiveness of evidence-based, online interventions for PPU using rigorous methods (Griffin et al., 2021; Grubbs et al., 2020; Grubbs & Kraus, 2021). We examined the feasibility and initial efficacy of an online PPU intervention using a randomized controlled trial study design (Rounsaville et al., 2001). Participants evaluated the intervention positively and reported significantly lower PPU and beneficial changes in other pornography use-related characteristics at the six-week follow-up, compared to the control group, indicating the potential effectiveness of the intervention. However, the attrition was high, especially in the intervention group, limiting the generalizability of the findings. Possible ways to strengthen the intervention were identified.

### Preliminary effectiveness of the intervention

The recruitment was successful, and the target sample size was reached within 23 months. Participants’ sociodemographic characteristics (e.g., gender, age, relationship status, sexual orientation) were similar to previous PPU treatment-seeking samples (Gola et al., 2016; Kraus et al., 2016; Lewczuk et al., 2017). On average, participants reported higher PPU scores than the recommended cut-off (Bőthe, Tóth-Király, et al., 2018), and reported high pornography use frequency (i.e., 45% of participants used pornography daily or more often) at baseline, suggesting that we had successfully identified the target population (Orsmond & Cohn, 2015). Concerning our primary outcome, in line with our hypothesis (Bőthe, Baumgartner, et al., 2020), PPU significantly decreased in the intervention group at the six-week follow-up, compared to the control group. Regarding the secondary outcomes, findings were in line with the hypothesized changes; participants in the intervention group reported significantly lower pornography use frequency, self-perceived pornography addiction and craving, and higher pornography avoidance self-efficacy at the six-week follow-up. However, participants’ moral incongruence toward pornography use and time spent with pornography use on each session did not change significantly, presumably as these topics were not as pronounced in the intervention as others. Although our analyses were exploratory, given the small sample size and low statistical power, our preliminary findings suggest that the intervention hold promise in reducing participants’ PPU (Bowen et al., 2009; Orsmond & Cohn, 2015; Rounsaville et al., 2001). Moreover, as our measures could detect the change in our primary and secondary outcomes, they provided evidence of being appropriate for the population and future studies (Orsmond & Cohn, 2015).

### Overall and differential attrition rates, adherence, and potential reasons of dropout

Although we expected high dropout rates based on the results of online intervention studies (Rooke, Copeland, Norberg, Hine, & McCambridge, 2013; Schaub et al., 2015), the attrition was especially high in the intervention group, limiting the findings’ generalizability. Specifically, 55% of the participants completed the six-week follow-up in the control group, while only 11% completed it in the intervention group. When examining overall attrition, participants who completed the follow-up assessment and dropped out reported similar initial levels of sociodemographic and psychological characteristics, except for satisfaction with life. Participants dropping out from the study had higher initial life satisfaction, suggesting that those participants were retained who might have experienced more problems in their life in general. Concerning differential attrition, dropout analysis revealed that participants who provided follow-up data did not differ significantly from dropouts in their baseline characteristics in the control group. However, in line with previous findings (Gottlieb, Horwitz, Kraus, Segal, & Viscoli, 1994; Panlilio et al., 2019), dropouts reported significantly higher pornography craving at baseline than participants who completed the intervention. As higher levels of dysregulated pornography use may be related to higher avoidant coping strategies (Lewczuk et al., 2020), for individuals with high pornography craving, participating in an online intervention that focuses on pornography use might be triggering. Thus, craving might play an important role in adherence and should be presented as soon as possible during the intervention. Another possibility might be to open all modules from the beginning of the intervention, or prepare personalized suggestions about the order of the modules for each participant based on their baseline survey results.

It is important to note that similar attrition rates were observed in PPU treatment-seeking men in a recent study. Similarly to our results, only 11% of the participants completed the follow-up survey after participating in an app-based intervention for PPU (Chen, Jiang, Luo, Kraus, & Bőthe, 2021). Based on follow-up interviews with participants dropping out from the study, several potential mechanisms were mentioned that might explain the high attrition, such as the unsuitability of reminders, feelings that pornography use-related problems did not change during the intervention, or using avoidance as a potential coping strategy (i.e., avoiding pornography-related content, including the follow-up surveys) (Chen et al., 2021). These explanations might apply to the present study as well. Moreover, the higher follow-up completion rate in the control group might result from the fact that participants in the control group were provided access to the intervention three months after completing the baseline survey. Therefore, these participants might have been more motivated to complete all surveys while waiting for the intervention, whereas participants in the intervention group did not have any incentives to complete the follow-up survey after receiving the intervention. However, future studies are needed to map other potential reasons for dropout.

When examining the adherence rate in the intervention group (Orsmond & Cohn, 2015), a gradual decrease can be observed in completion from module to module (from 66% to 12%). Therefore, we examined potential differences between participants in the intervention group who did not complete any modules, completed only one module, and completed more than one module. Participants were significantly younger in the group of those who only completed one module, compared to those who did not complete any modules and completed more than one module. However, no other significant differences were observed in participants’ sociodemographic, pornography use-related, or psychosocial characteristics. These findings suggest that no systematic attrition might have been present in the study (e.g., more severe cases dropping out of the study before completing any modules, or after completing only one module).

The results of the present study align with previous online interventions’ findings concerning low adherence rates (Amann et al., 2018), presumably resulting from the absence of incentives. Thus, future studies should provide incentives for participants. Nevertheless, it should be noted that funding agencies usually demonstrate little interest in supporting pornography use research, limiting researchers’ resources (Grubbs & Kraus, 2021). Moreover, low adherence might also derive from the specific features of online interventions, such as the absence of personal relationships and personalized exercises, and participants receiving only email invitations to complete the follow-up surveys (Amann et al., 2018; Beintner, Emmerich, Vollert, Taylor, & Jacobi, 2019). Although higher adherence might be achieved by calls or text message reminders, it would violate anonymity, which can be especially important for individuals with PPU who might experience great shame and guilt concerning their pornography use (Sniewski & Farvid, 2020). This notion was supported by the fact that several participants used throwaway email accounts, corroborating participants’ need for anonymity.

### Participants’ feedback and potential changes in the intervention

Participants evaluated the intervention positively concerning its usefulness, understandability, appearance, and length, illustrating the acceptability of the intervention (Bowen et al., 2009). These findings corroborated the results of previous online interventions, suggesting that evidence-based, online self-help materials may reduce treatment barriers and provide free and easy-to-use tools to reduce problematic behaviors (Baumgartner et al., 2019; Haug et al., 2018; Herrero et al., 2019; Weisel et al., 2018). Still, based on participants’ feedback, some changes in study design and content of intervention are suggested for future research.

Participants found the craving and success preservation modules the most useful, further supporting the importance of focusing on craving in the first modules of the intervention. Participants were satisfied with the length of the modules in general. However, reported that the “how to feel better without pornography” module was too long. Yet, it needs to be noted that some participants find it helpful that several activity lists were included in this module. Thus, restructuring and reducing the length of this module might be beneficial, with keeping the activity lists. Participants found all modules understandable and were satisfied with their appearance. Still, they suggested adapting the intervention to a more mobile-friendly format (e.g., smaller figures), adding more explanation and specific examples to some tasks (e.g., previous successful efforts to change problematic behaviors), putting more emphasis on masturbation, and reducing the number of questions, typing tasks, and materials to read.

Other potential improvement targets may involve adding more companions to the intervention relate (e.g., older companions), as some participants did not find any companion to whom they could. In line with previous findings (Amann et al., 2018), some participants missed human interaction. Future studies may complement this intervention with social support, or guidance teams found effective in previous online interventions (Baumgartner et al., 2021; Zarski et al., 2016). The suggested changes for improving Hands-off may increase its potential effectiveness in future studies, and the findings extended our insights in designing effective online interventions for PPU in general.

### Limitations and future directions

The majority of participants lived in the US, England, or Canada; were heterosexual men; were highly educated (i.e., had a college or university degree); and had a high socioeconomic status (i.e., the sample was Western, educated, industrialized, rich, and democratic, WEIRD), limiting the generalizability of the findings. Further examination is needed to examine the potential efficacy of the intervention in other non-WEIRD populations (Griffin et al., 2021; Klein et al., 2021). The high dropout rate, low adherence, and differences between those who completed and did not complete the follow-up assessment (e.g., higher levels of craving among those who dropped out from the intervention group) might also limit the conclusions drawn from our findings. As discussed above, several strategies (e.g., incentives) should be applied to retain higher adherence rates, providing the opportunity for more comprehensive analyses (e.g., including sexual wellbeing or general wellbeing, or other mental health-related variables). Lastly, no objective criteria were used to exclude participants from the study who had not demonstrated dysregulated, compulsive, or problematic pornography use, as the intervention was designed to be potentially effective in all groups of treatment-seeking individuals (e.g., individuals with dysregulated pornography use or individuals with self-perceived PPU due to moral incongruence towards pornography use) (Chen et al., 2021; Grubbs, Perry, Wilt, & Reid, 2019; Kraus & Sweeney, 2019). Therefore, it is possible that some individuals might have realized that their pornography use is not problematic or might have simply been interested in the content of the intervention without any objective or self-perceived PPU, resulting in high dropout rates. However, the number of these individuals should be low, as no significant differences were be observed in the baseline levels of PPU, self-perceived pornography addiction, and moral incongruence towards pornography use of individuals in the intervention group who did not complete any modules, completed only one module, or completed more than one module.

Our preliminary findings show potential for PPU treatment and provide a basis for future adequately powered randomized controlled trials following previously established guidelines and recommendations of evaluating the feasibility (stage I), effectiveness (stage II), and transportability (stage III) of new interventions (Rounsaville et al., 2001). As a next step (stage II), studies should evaluate the effectiveness of the Hands-off intervention using an adequately powered RCT study design with one or more active control conditions (e.g., comparing the intervention to traditional, offline treatment; self-help groups; or self-help books or online applications) (Becker, Haug, Sullivan, & Schaub, 2014; Rounsaville et al., 2001; Weisel et al., 2019). Moreover, addressing the potential mechanisms underlying the effectiveness of the treatment (i.e., theory of change mechanisms, why and how the intervention is effective) would be beneficial in this step, especially as most previous studies on PPU have been conducted without strong theoretical models and integrating the understandings of PPU into larger theoretical frameworks (e.g., network models of psychopathologies) (Bőthe, Lonza, Štulhofer, & Demetrovics, 2020; Grubbs et al., 2020; Rounsaville et al., 2001; Werner, Štulhofer, Waldorp, & Jurin, 2018).

After establishing the effectiveness of the intervention in at least two RCTs, an essential next step is the examination of the transportability of the intervention to different settings (stage III) (Rounsaville et al., 2001). For example, one key issue would be the evaluation of the generalizability of the intervention’s effectiveness in non-WEIRD populations, among women and non-binary individuals, other cultures, or other languages (Griffin et al., 2021; Klein et al., 2021). Moreover, individuals’ treatment-seeking for PPU may derive from actual behavioral dysregulation (i.e., PPU), moral incongruence towards pornography use (i.e., self-perceived PPU due to moral incongruence towards pornography use), or both (i.e., behavioral dysregulation and moral incongruence towards pornography use) (Grubbs et al., 2019; Grubbs & Perry, 2019; Kraus & Sweeney, 2019). Therefore, the intervention’s effectiveness should be tested in all these populations. The intervention might also be adapted to meet the different treatment needs and goals of these populations (e.g., participants might be allocated to the most appropriate version of the intervention based on the results of their baseline survey, following a decision tree or algorithm of differential diagnosis and treatment) (Kraus & Sweeney, 2019). Following the recommendations of the stage model of behavioral therapies research may help facilitate bridging the gap between research findings and clinical practice, and providing guidelines for choosing the most optimal treatment options for individuals with PPU (Rounsaville et al., 2001).

## Conclusions

Preliminary results suggested that participants in the intervention group reported lower PPU, self-perceived pornography addiction, pornography use frequency, pornography craving, and better pornography avoidance self-efficacy compared to the control group. Although the present study was a first step (i.e., feasibility study) in rigorous treatment studies for compulsive sexual behaviors and PPU (Griffin et al., 2021; Grubbs et al., 2020; Grubbs & Kraus, 2021; Rounsaville et al., 2001), findings are promising that online interventions for PPU might help reduce PPU in some cases, reducing treatment barriers.

## Funding

The research was supported by the Hungarian National Research, Development, and Innovation Office (Grant numbers: KKP126835, NKFIH-1157-8/2019-DT, K134807). BB was supported by the ÚNKP-18-3 New National Excellence Program of the Ministry of Human Capacities to develop the intervention protocol. BB was supported by the Merit Scholarship Program for Foreign Students (PBEEE) awarded by the Ministère de l’Éducation et de l’Enseignement Supérieur (MEES) and by a postdoctoral fellowship from the SCOUP Team – Sexuality and Couples – Fonds de recherche du Québec, Société et Culture during the finalization of the paper.

## Authors’ contribution

BB, CB, MPS, ZD, and GO set up the initial idea and plan for this study. BB and CB prepared the first draft of the paper and BB finished the final manuscript. BB and GO developed the intervention of study arm 1. CB, MPS, and ZD helped throughout the development of the intervention. CB programmed and implemented the intervention website. CB performed statistical analysis. GO, MPS, and ZD and gave valuable feedback to the study. All authors approved the final version of the manuscript submitted for publication. BB is the guarantor.

## Conflict of interest

The authors declare no conflict of interest.

## Pre-registration

The study was preregistered on the Open Science Framework (OSF) Website: https://osf.io/5tqkb/

## Footnotes

1 Given high attrition rates, we needed to deviate from the analysis plan published in the study protocol paper (Bőthe, Baumgartner, et al., 2020) and focused only on pornography use-related characteristics.

2 As pornography use frequency was assessed on an ordinal scale, we recoded this variable in the analyses: “weekly” = 1; “2–3 times a week” = 2.5; “4–5 times a week” = 4.5; “6–7 times a week” = 6.5; “more than 7 times a week” = 7.5.

## Appendix 1

**Appendix 1.** Consort 2010 checklist of information to include when reporting a randomised trial*

Section/Topic	Item No	Checklist item	Reported on page No
*Title and abstract*			
	1a	Identification as a randomised trial in the title	1
	1b	Structured summary of trial design, methods, results, and conclusions (for specific guidance see CONSORT for abstracts)	1
*Introduction*			
Background and objectives	2a	Scientific background and explanation of rationale	2
	2b	Specific objectives or hypotheses	2
*Methods*			
Trial design	3a	Description of trial design (such as parallel, factorial) including allocation ratio	3–5
	3b	Important changes to methods after trial commencement (such as eligibility criteria), with reasons	NA
Participants	4a	Eligibility criteria for participants	3
	4b	Settings and locations where the data were collected	3
Interventions	5	The interventions for each group with sufficient details to allow replication, including how and when they were actually administered	3–5
Outcomes	6a	Completely defined pre-specified primary and secondary outcome measures, including how and when they were assessed	5–6
	6b	Any changes to trial outcomes after the trial commenced, with reasons	2, 6
Sample size	7a	How sample size was determined	3
	7b	When applicable, explanation of any interim analyses and stopping guidelines	NA
*Randomisation:*			
Sequence generation	8a	Method used to generate the random allocation sequence	3
	8b	Type of randomisation; details of any restriction (such as blocking and block size)	3
Allocation concealment mechanism	9	Mechanism used to implement the random allocation sequence (such as sequentially numbered containers), describing any steps taken to conceal the sequence until interventions were assigned	3
Implementation	10	Who generated the random allocation sequence, who enrolled participants, and who assigned participants to interventions	3
Blinding	11a	If done, who was blinded after assignment to interventions (for example, participants, care providers, those assessing outcomes) and how	3
	11b	If relevant, description of the similarity of interventions	NA
Statistical methods	12a	Statistical methods used to compare groups for primary and secondary outcomes	6
	12b	Methods for additional analyses, such as subgroup analyses and adjusted analyses	6
*Results*			
Participant flow (a diagram is strongly recommended)	13a	For each group, the numbers of participants who were randomly assigned, received intended treatment, and were analysed for the primary outcome	3–6
	13b	For each group, losses and exclusions after randomisation, together with reasons	3
Recruitment	14a	Dates defining the periods of recruitment and follow-up	3
	14b	Why the trial ended or was stopped	3
Baseline data	15	A table showing baseline demographic and clinical characteristics for each group	4–5
Numbers analysed	16	For each group, number of participants (denominator) included in each analysis and whether the analysis was by original assigned groups	8
Outcomes and estimation	17a	For each primary and secondary outcome, results for each group, and the estimated effect size and its precision (such as 95% confidence interval)	7–8
	17b	For binary outcomes, presentation of both absolute and relative effect sizes is recommended	NA
Ancillary analyses	18	Results of any other analyses performed, including subgroup analyses and adjusted analyses, distinguishing pre-specified from exploratory	7–11, 19–21
Harms	19	All important harms or unintended effects in each group (for specific guidance see CONSORT for harms)	NA
*Discussion*			
Limitations	20	Trial limitations, addressing sources of potential bias, imprecision, and, if relevant, multiplicity of analyses	13
Generalisability	21	Generalisability (external validity, applicability) of the trial findings	11–13
Interpretation	22	Interpretation consistent with results, balancing benefits and harms, and considering other relevant evidence	11–13
*Other information*			
Registration	23	Registration number and name of trial registry	14
Protocol	24	Where the full trial protocol can be accessed, if available	3, 14
Funding	25	Sources of funding and other support (such as supply of drugs), role of funders	14

*We strongly recommend reading this statement in conjunction with the CONSORT 2010 Explanation and Elaboration for important clarifications on all the items. If relevant, we also recommend reading CONSORT extensions for cluster randomised trials, non-inferiority and equivalence trials, non-pharmacological treatments, herbal interventions, and pragmatic trials. Additional extensions are forthcoming: for those and for up to date references relevant to this checklist, see www.consort-statement.org.

## Appendix 2

**Table A1.** Baseline sociodemographic, pornography use-related, and psychological characteristics of participants in the intervention group who did not complete any modules, completed only one module, and completed more than one module

	Did not complete any moules (*n* = 41)	Completed only one module (*n* = 37)	Completed more than one module (*n* = 45)	*Statistical Analysis (Chi-Square Test, ANOVA)*
*Gender*, *n* (%)				
Woman	4 (9.8)	0 (0)	1 (2.2)	*NA*
Man	37 (90.2)	37 (100.0)	44 (97.8)	
*Age*, *M (SD)*	35.4 (11.7)	28.4 (8.8)	35.4 (12.2)	*F* (2,120) = 5.14, *P* = 0.007
*Highest education*, *n* (%)				*χ* ^2^ (2, *N* = 123) = 3.28, *P* = 0.194
Primary school	0 (0.0)	0 (0.0)	0 (0.0)	
Vocational school	1 (2.4)	1 (2.7)	0 (0.0)	
High school	12 (29.3)	7 (18.9)	7 (15.6)	
College or university	28 (68.3)	29 (78.4)	38 (84.4)	
*Country of origin*, *n* (%)				*χ* ^2^ (10, *N* = 123) = 10.11, *P* = 0.431
United States	13 (31.7)	13 (35.1)	17 (37.8)	
England	6 (14.6)	5 (13.5)	8 (17.8)	
Canada	5 (12.2)	2 (5.4)	5 (11.1)	
Hungary	5 (12.2)	3 (8.1)	2 (4.4)	
India	0 (0.0)	3 (8.1)	0 (0.0)	
Other (combined^a^)	12 (29.3)	11 (29.7)	13 (28.9)	
*Relationship status*, *n* (%)				*χ* ^2^ (8, *N* = 123) = 14.94, *P* = 0.060
Single	15 (36.6)	17 (45.9)	11 (24.4)	
In a relationship	12 (29.3)	16 (43.2)	12 (26.7)	
Married	13 (31.7)	4 (10.8)	20 (44.4)	
Engaged	1 (2.4)	0 (0.0)	1 (2.2)	
Other	0 (0.0)	0 (0.0)	1 (0.0)	
*Sexual orientation, n* (%)				*χ* ^2^ (6, *N* = 123) = 5.54, *P* = 0.477
Heterosexual	31 (75.6)	27 (73.0)	34 (75.6)	
Homosexual	1 (2.4)	4 (10.8)	2 (4.4)	
Bisexual	6 (14.6)	3 (8.1)	8 (17.8)	
Unsure	3 (7.3)	3 (8.1)	1 (2.2)	
*Sought treatment for pornography use previously, n* (%)	13 (31.7)	12 (32.4)	15 (33.3)	*χ* ^2^ (2, *N* = 123) = 0.02, *P* = 0.987
*Problematic pornography use* (Range 0–126), *M (SD)*	76.5 (19.8)	82.5 (13.7)	82.4 (15.4)	*F* (2,120) = 1.74, *P* = 0.180
*Pornography use frequency*, *n* (%)				*χ* ^2^ (2, *N* = 123) = 1.23, *P* = 0.541
>7 a week	14 (34.1)	9 (24.3)	12 (26.7)	
6–7 a week	7 (17.1)	7 (18.9)	7 (15.6)	
4–5 a week	8 (19.5)	7 (18.9)	7 (15.6)	
2–3 a week	5 (12.2)	6 (16.2)	8 (17.8)	
weekly	2 (4.9)	3 (8.1)	6 (13.3)	
Less frequently	5 (12.2)	5 (13.5)	5 (11.1)	
*Time spent with pornography use per session in minutes*, *M (SD)*	46.9 (44.1)	47.4 (35.9)	57.7 (57.8)	*F* (2,120) = 0.70, *P* = 0.499
*Moral incongruence concerning pornography use* (Range 0–6), *M (SD)*	2.7 (1.9)	3.2 (2.2)	3.2 (2.2)	*F* (2,120) = 0.81, *P* = 0.447
*Self-perceived pornography addiction* (Range 0–6), *M (SD)*	4.5 (1.7)	4.7 (1.5)	4.7 (1.2)	*F* (2,120) = 0.42, *P* = 0.656
*Pornography craving* (Range 0–60), *M (SD)*	42.9 (16.2)	50.5 (14.7)	46.9 (16.1)	*F* (2,120) = 0.04, *P* = 0.109
*Pornography avoidance self-efficacy* (Range 0–100), *M (SD)*	54.1 (20.1)	49.1 (14.5)	53.1 (18.8)	*F* (2,120) = 0.85, *P* = 0.432
*Sex mindset*, (Range 5–30), *M (SD)*	13.5 (5.1)	12.5 (5.5)	13.0 (5.1)	*F* (2,120) = 0.32, *P* = 0.726
*Sexual satisfaction,* (Range 0–4), *M (SD)*	2.3 (1.4)	1.9 (1.4)	2.1 (1.2)	*F* (2,77) = 0.58, *P* = 0.564
*Satisfaction with life,* (Range 5–35), *M (SD)*	18.5 (7.6)	17.2 (7.0)	20.7 (7.5)	*F* (2,120) = 2.36, *P* = 0.098
*Self-report adult ADHD* ^b^ (Range 0–24), *M (SD)*	11.2 (4.3)	12.7 (3.7)	12.0 (4.5)	*F* (2,120) = 1.30, *P* = 0.276
*Alcohol-related problems*, (Range 0–45), *M (SD)*	3.5 (7.6)	2.8 (6.3)	1.6 (3.1)	*F* (2,120) = 1.13, *P* = 0.328
*Psychiatric symptoms (depressive, anxiety, and somatization symptoms)* (Range 0–72), *M (SD)*	15.9 (11.7)	22.1 (13.3)	17.5 (12.1)	*F* (2,120) = 2.59, *P* = 0.079
*Positive emotions,* (Range 0–20), *M (SD)*	14.6 (3.6)	14.9 (3.8)	15.5 (3.2)	*F* (2,120) = 0.68, *P* = 0.508
*Negative emotions,* (Range 0–20), *M (SD)*	17.5 (3.9)	17.2 (4.5)	17.1 (3.8)	*F* (2,120) = 0.15, *P* = 0.864
*Cannabis use* ^*c*^, *n* (%)				
Never	23 (56.1)	26 (70.3)	35 (77.8)	*χ* ^2^ (4, *N* = 123) = 5.52, *P* = 0.063
Once or twice	8 (19.5)	5 (16.2)	6 (13.3)	
Monthly	2 (4.9)	2 (5.4)	0 (0)	
Weekly	3 (7.3)	3 (8.1)	3 (6.7)	
Daily or almost daily	5 (12.2)	0 (0.0)	1 (2.2)	
*Prescription stimulants use* ^*c*^, *n* (%)				
Never	35 (85.4)	30 (81.1)	39 (86.7)	*χ* ^2^ (4, *N* = 123) = 0.67, *P* = 0.716
Once or twice	0 (0.0)	0 (0.0)	2 (4.4)	
Monthly	0 (0.0	0 (0.0)	0 (0.0)	
Weekly	1 (2.4)	0 (0.0)	0 (0.0)	
Daily or almost daily	5 (12.2)	7 (18.9)	4 (8.9)	

*Note*. ^a^Less than 5 participants in each unlisted country, ^b^ADHD: attention deficit hyperactivity disorder, ^c^Previous three months; the two most commonly used substances (i.e., cannabis and prescription stimulants) are included in table. *M* = mean, *SD* = standard deviation, *NA* = not applicable.
